# Burial or cremation? Factors associated with preferences among patients with cancer in Brazil: a cross-sectional study

**DOI:** 10.1590/1516-3180.2022.0441.R1.13022023

**Published:** 2023-05-12

**Authors:** Bianca Sakamoto Ribeiro Paiva, Bruna Minto Lourenço, Henrique Moraes Prata, Talita Caroline de Oliveira Valentino, Marco Antonio de Oliveira, Martins Fideles dos Santos, Eduardo Bruera, Carlos Eduardo Paiva

**Affiliations:** I PhD. Nurse, Researcher and Professor, Oncology Graduate Program, Grupo de Pesquisa em Cuidados Paliativos e Qualidade de Vida Relacionada à Saúde (GPQual), Hospital de Câncer de Barretos, Barretos (SP), Brazil.; II BM. Undergraduate Psychology Student, Oncology Graduate Program, Grupo de Pesquisa em Cuidados Paliativos e Qualidade de Vida Relacionada à Saúde (GPQual), Hospital de Câncer de Barretos, Barretos (SP), Brazil.; III PhD. Lawyer, Oncology Graduate Program, Grupo de Pesquisa em Cuidados Paliativos e Qualidade de Vida Relacionada à Saúde (GPQual), Hospital de Câncer de Barretos, Barretos (SP), Brazil.; IV Doctoral Student and Nurse, Oncology Graduate Program, Grupo de Pesquisa em Cuidados Paliativos e Qualidade de Vida Relacionada à Saúde (GPQual), Hospital de Câncer de Barretos, Barretos (SP), Brazil.; V MSc. Biostatistics, Learning and Research Institute, Grupo de Pesquisa em Cuidados Paliativos e Qualidade de Vida Relacionada à Saúde (GPQual), Hospital de Câncer de Barretos, Barretos (SP), Brazil.; VI MSc. Librarian, Oncology Graduate Program, Faculdade de Ciências da Saúde de Barretos Dr. Paulo Prata (FACISB), Barretos (SP), Brazil; Grupo de Pesquisa em Cuidados Paliativos e Qualidade de Vida Relacionada à Saúde (GPQual), Hospital de Câncer de Barretos, Barretos (SP), Brazil.; Hospital de Câncer de Barretos, Grupo de Pesquisa em Cuidados Paliativos e Qualidade de Vida Relacionada à Saúde (GPQual), Barretos, SP, Brazil; VII MD. Physician, Department of Palliative, Rehabilitation, and Integrative Medicine, Division of Cancer Medicine, The University of Texas MD Anderson Cancer Center, Houston (TX), United States.; VIII PhD. Physician and Professor, Postgraduation, Grupo de Pesquisa em Cuidados Paliativos e Qualidade de Vida Relacionada à Saúde (GPQual), Hospital de Câncer de Barretos, Barretos (SP), Brazil.

**Keywords:** Death, Cremation, Burial, Preferences, Burial and factors related, End of life, Oncology

## Abstract

**BACKGROUND:**

People living with life-limiting illnesses and their family caregivers consistently emphasize the importance of preparing for imminent death, with planned funerals being a common aspect of this preparation. Few studies have described the funeral rituals or post-mortem preferences of patients with cancer.

**OBJECTIVE:**

To evaluate the percentage of patients with cancer who wish to be cremated and to identify the factors associated with this preference.

**DESIGN AND SETTING:**

Cross-sectional study conducted at Barretos Cancer Hospital.

**METHODS:**

A total of 220 patients with cancer completed a Sociodemographic and Clinical Questionnaire, the Duke University Religiosity Index, and burial or cremation preferences. Binary Logistic Regression was performed to identify independent variables associated with cremation.

**RESULTS:**

Of the 220 patients, 25.0% preferred cremation and 71.4% preferred burial. Talks about death with family or close friends in their daily life (odds ratio, OR = 2.89; P = 0.021), patients that answered “other” (unsure, tends not be true and not true) for religious beliefs are what really lie behind my whole approach to life (OR = 20.34; P = 0.005), and education 9 to 11 years (OR = 3.15; P = 0.019) or ≥ 12 years (OR = 3.18; P = 0.024) were associated with cremation preference.

**CONCLUSION:**

Most patients with Cancer in Brazil prefer burial after death. Discussions about death, religious beliefs and involvement, and educational level seem to influence the preference for cremation. A deeper understanding of ritual funeral preferences and their associated factors may guide policies, services, and health teams in promoting the quality of dying and death.

## INTRODUCTION

People living with life-limiting illnesses and their family caregivers emphasize the importance of preparing for imminent death, and planning funeral rituals is a common aspect of this preparation. The discussion of funeral ritual preferences may be challenging in many cultures.^1,2^ Funeral rituals are technical actions of dead body preparation, display, and burial or cremation, considering symbolic acts that change according to the culture of the people.^3^ Cremation aims to reduce a body to ashes by burning it and these ashes are given to the family.^4–6^

Few studies have described the funeral ritual preferences of patients with cancer, including those in Brazil, and have not explored the factors that may be related to these preferences.^7^ Identifying them can provide guidance to those providing care (either professionally or voluntarily) to improve the end-of-life process of patients. Such fulfilment of patients' wishes can improve the quality of death of the patients and the grieving process of loved ones.

## OBJECTIVE

This study aims to evaluate the percentage of patients with cancer who wish to undergo cremation and identify the factors associated with this preference.

## METHODS

### Study design and place

This cross-sectional descriptive study was performed from August/2021 to March/2022 at Barretos Cancer Hospital (Sao Paulo, Brazil).

### Participants

Patients from the oncology outpatient clinic and chemotherapy infusion center were invited to participate. Eligibility criteria included ≥18-year-old, cancer diagnosis, undergoing individual or concomitant treatment of chemotherapy, surgery, radiotherapy or hormone therapy, cognitive capacity and coherent communication, no acute psychiatric illness, and no recent medical communication of bad news.

### Data collection

This study was approved by the Research Ethics Committee of the Barretos Cancer Hospital (No. 4.312.986; date: October 1, 2020). Interviews were conducted *face-to-face* after the participants answered the sociodemographic and clinical information questionnaires. Participants were also invited to fill in the Duke Religion Index, a questionnaire that measures religious beliefs and involvement.^8^ The patients’ attitudes and beliefs regarding cremation and burial were also determined by the research team, developing a survey based on the literature to obtain information regarding funeral ritual preferences in the cultural context.^9–11^ The clarity and pertinence of each item of the Burial and Cremation Preference Survey was evaluated by a committee of experts.^12^ Data were recorded using Research Electronic Data Capture (REDCap).^13^

### Statistic

The sample size was calculated based on prevalence estimates. For this purpose, it was considered that the cremation rates of Colombia and Argentina in 2017 ranged from 2.1% to 25.4%^14^ and, in Brazil, it was approximately 10%, with a precision of 4% and a 95% confidence interval.^15^ The minimum sample size was 216 participants.

Descriptive statistics were used to summarize patient characteristics. Chi-square or Fisher's exact test, t-test, or Mann-Whitney U test were used to examine the difference between patient characteristics and ritual funeral preference (cremation: yes versus no). To identify independent predictors associated with funeral ritual preference, variables (P < 0.20 were included in the initial Binary Logistic Regression Model. For the final model adjustment, the variables were selected using the backward method, and the model comprised variables with P < 0.05. Multicollinearity was verified by estimating variance inflation factors (VIF).

Data were analyzed by IBM-SPSS v.27.0 (IBM Corp., Armonk, New York, United States). Statistical significance was set at P < 0.05, considered significant.

## RESULTS

A total of 220 (48.5%) of the 454 eligible patients were included in the study. A total of 234 patients were excluded because of recent medical communication of bad news (n = 133; 57.0%), refusal (n = 82; 35.0%), or the absence of full cognitive capacity (n = 19; 8.0%). The main reasons expressed by patients who refused to participate in the study were feeling uncomfortable talking about death (n = 48; 58.5%), absence of interest in participating in the study (n = 30; 36.6%), and the presence of uncontrolled symptoms at the time of the approach (n = 4;4.9%).

The mean age was 51.8 years; 167 (75.9%) patients were female; 114 (51.8%) were white, 146 (66.4%) were married/with partner, and 85 (38.7%) had a low educational level. The most common types of cancer were breast (n = 113; 51.4%) and gastrointestinal (n = 62; 28.2%). Overall, funeral ritual preferences were burial (n = 157; 71.4%), cremation (n = 55; 25.0%), and indifference (n = 8; 3.6%).

Univariate analysis identified the variables associated with ritual funeral preferences. These variables included the patient's age, ethnicity, education, human development index of the city of origin, self-perception of health, talking about death with one's family or close friends, talking about one's wishes regarding one's own funeral, and considering cremation as an easier alternative if there were difficulties in transporting the body and paying for this process (Table 1).

**Table 1:** Association between demographic and clinical characteristics and religious involvement with ritual funeral preference of cancer patients

Variables				Cremation		P value
				No n (%)	Yes n (%)	
	Demographic characteristics					
		**Age** Years, average (SD)		52.2 (13.0)	51.3 (12.4)	**0.003** **
		**Gender**	Male	37 (23.6)	14 (25.5)	0.855
			Female	120 (76.4)	41 (74.5)	
		**Ethnicity**	White	73 (46.5)	39 (70.9)	**0.014** *
			Black	17 (10.8)	4 (7.3)	
			Brown	64 (40.8)	11 (20.0)	
			Yellow	3 (1.9)	1 (1.8)	
		**Education**	0 to 8 years	72 (45.9)	12 (21.8)	**0.006**
			9 to 11 years	47 (29.9)	21 (38.2)	
			≥ 12 years	38 (24.2)	22 (40.0)	
		**Family income**	No income	3 (1.9)	4 (7.3)	**0.002** *
			1 to 3 minimum wages	117 (74.5)	28 (50.9)	
			4 to 6 minimum wages	28 (17.8)	13 (23.6)	
			≥ 7 minimum wages	9 (5.7)	10 (18.2)	
		**HDI of city of origin**	Low	10 (6.4)	1 (1.8)	**0.004** *
			Medium	32 (20.4)	7 (12.7)	
			High	108 (68.8)	36 (65.5)	
			Very high	7 (4.5)	11 (20.0)	
	**Clinical characteristics**					
		**Type of cancer**	Breast	76 (48.4)	32 (58.2)	0.768*
			Gastrointestinal	46 (29.3)	14 (25.5)	
			Others	35 (22.3)	9 (16.4)	
		**Health self-perception**	Very good	17 (10.8)	13 (23.6)	**0.081** *
			Good	79 (50.3)	28 (50.9)	
			Regular	56 (35.7)	13 (23.6)	
			Poor	5 (3.2)	1 (1.8)	
	**Duke Religion Index**					
		**How often do you attend church or other religious Meetings**	More than once/week	99 (63.1)	32 (58.2)	0.864*
			Other frequency^1^	58 (36.9)	23 (41.8)	
		**How often do you spend time in private religious activities, such as prayer, meditation or bible study**	More than once a day	132 (84.1)	47 (85.5)	0.936
			Other frequency^2^	25 (15.9)	8 (14.5)	
		**In my life, I experience the presence of the God or Holy Spirit**	Totally true for me/true	157 (100.0)	53 (96.4)	**0.133** *
			Other (in general not true)^3^	0 (0.0)	2 (3.6)	
		**My religious beliefs are what really lie behind my whole approach to life**	Totally true for me/true	154 (98.1)	51 (92.7)	**0.152**
			Other (in general not true)^3^	3 (1.9)	4 (7.3)	
		**I try hard to carry my religion over into all other dealings in life**	Totally true for me/true	149 (94.9)	51 (92.7)	0.692*
			Other (in general not true)^3^	8 (5.1)	4 (7.3)	
	**Burial and Cremation Preference Questionnaire**					
		**Talks about death with your family or close friends**	No	64 (40.8)	10 (18.2)	**0.003**
			Yes	93 (59.2)	45 (81.2)	
		**Talks about your wishes regarding own funeral**	No	98 (62.4)	20 (36.4)	**0.001**
			Yes	59 (37.6)	35 (63.6)	
	**If you know what cremation is:**					
		**Manifested the wish to be cremated by a loved one**	No	2 (25.0)	15 (27.8)	1.000*
			Yes	6 (75.0)	39 (72.2)	
	**Greater difficulty for desire to be cremated not being fulfilled**					
		**Cremation cost is very expensive**	No	2 (25.0)	19 (35.2)	0.705*
			Yes	6 (75.0)	35 (64.8)	
		**My family not accept cremation**	No	7 (87.5)	46 (85.2)	1.000*
			Yes	1 (12.5)	8 (14.8)	
		**My religion not approve of cremation**	No	8 (100.0)	50 (92.6)	1.000*
			Yes	0 (0.0)	4 (7.4)	
		**There is no crematorium in the city/near homes**	No	3 (37.5)	29 (53.7)	0.467*
			Yes	5 (62. 5)	25 (46.3)	
		**It's not common for people in my family to be cremated**	No	3 (37.5)	30 (55.6)	0.456*
			Yes	5 (62.5)	24 (44.4)	
		**Considers cremation as an easier alternative if there were difficulties in transporting the body and paying for this process**	No	62 (39.5)	6 (10.9)	**<0.001**
			Yes	95 (60.5)	49 (89.1)	

SD = standard deviation; HDI = human development index. Pearson's Chi-square test;

* Fisher Exact test;

** Man-Whitney test. P value 0.05. The option “yes” refers to patients who preferred to be cremated (n = 55) and the option “no” are those who preferred to be burial.

1 Other: Two to three times/month, a few times a year, once a year or less and never;

2 Other: two or more times/week, once a week, a few times/month and rarely or never;

3 Other: unsure, tends not be true and not true.

Table 2 reports the results of the Binary logistic regression analysis. Education 9 to 11 years (odds ratio, OR = 3.15; P = 0.019) or ≥ 12 years (OR = 3.18; P = 0.024), talks about death with family or close friends in their daily life (OR = 2.89; P = 0.021), and patients that answered “other” (unsure, tends not be true and not true) for religious beliefs are what really lie behind my whole approach to life (OR = 20.34; P = 0.005) were potential predictors associated with higher chances of cremation preference.

**Table 2:** Binary logistic regression analysis of the potential predictors associated with funeral ritual preference (cremation) in patients with cancer

Variable				Cremation (yes)			
				n (events)	Category	OR (IC 95%)	P value
**Demographic characteristic**							
	**Education**						
		0 to 8 years		84 (12)	1	–	–
		9 to 11 years		68 (21)		3.15 (1.21–8.24)	0.019
		≥ 12 years		60 (22)		3.18 (1.16–8.67)	0.024
	**HDI city of origin**						
		Very high		18 (11)	1	–	–
		High		144 (36)		0.18 (0.05–0.63)	0.007
		Medium		39 (7)		0.15 (0.03–0.67)	0.014
		Low		11 (1)		0.08 (0.00–1.00)	0.051
**Clinical characteristics**							
	**Health self-perception**						
		Very good		30 (13)	1	–	–
		Good		107 (28)		0.26 (0.09–0.75)	0.013
		Regular		69 (13)		0.18 (0.05–0.59)	0.005
		Poor		6 (1)		0.18 (0.01–2.61)	0.209
**Duke Religion Index**							
	**My religious beliefs are what really lie behind my whole**						
		**Approach to life**					
			Totally true for me/true	205 (51)	1	–	–
			Other (in general not true)^1^	7 (4)		20.34 (2.44–169.38)	0.005
**Burial and Cremation Preference Questionnaire**							
	**Talks about death with your family or close friends**						
		No		74 (10)	1	–	–
		Yes		138 (45)		2.89 (1.17–7.13)	0.021

Binary Logistic Regression Model; P value < 0.05 Wald test.

1 Other: unsure, tends not be true and not true.

OR = odds ratio; CI = confidence interval.

## DISCUSSION

In our study, the vast majority (71.4%) of patients preferred to be buried. Cremation was preferred by 25.0% of the patients. The findings may provide important information for the evaluation of profiles of patients who prefer cremation, and how health care professionals may help these patients realize their desires.

Religious teachings, traditions, beliefs, and education level may have an important influence on a patient's decision making about end-of-life care.^2^ The growing practice of cremation has provided many countries with a spread of locations offering this service, and made it cheaper as compared to burial.^16,17^ In many Asian cities with scarce physical space, funeral planning agencies have sought to reduce space for the dead by encouraging conversion from burial to cremation over several decades.^17^ In 2017, the cremation rate in Canada was 70.5%.^18^ Cremation rates are low in countries where Catholicism predominates.^19^ In the United States, meanwhile, the proportion of deceased persons who were cremated increased from 3.6% in 1960 to 48.6% in 2015, with a projected 71% by 2030.^20^

In Brazil, as the practice of cremation is not widespread, the funeral process and the location where cremation takes place still make choosing this method less feasible. This could be identified in our study, in which many participants did not choose cremation, justifying that the cost is too expensive or that the place that offers cremation services is located in cities far away from where they live. On the other hand, the alternative of cremation as a way to minimize situations in which there were difficulties and costs for the transfer of the body over long distances was an option mentioned by a good part of the patients.

Since talking about death or preparing for the moment of death is not in habit,^21^ it may hinder communication about terminality and opportunity for the patient to express their wishes about the funeral ceremony. In this study, not discussing the subject was motivated by the fact that the participants’ families did not have a culture of this dialogue.

This study had some limitations. First, it was cross-sectional, and it was, therefore, impossible to determine cause-and-effect relationships. Second, it was conducted at a single reference center of oncology in Brazil, which provides care to patients in different regions of the country. Third, most participants were very religious; that is, it was not possible to identify a sample of nonreligious patients for comparison. Other studies have found that patients with advanced cancer express a high frequency of religiosity.^22^ There was an important number of patients not agreeing to participate in the research, which may be a sampling bias. It is possible that these patients experienced greater stigma about death and preferences for more traditional funeral methods in Brazil.

## CONCLUSION

Most Brazilian patients with cancer prefer burial after death. Discussions about death, religious beliefs and involvement, and educational level seem to influence the preference for cremation. A deeper understanding of ritual funeral preferences and their associated factors may guide policies, services, and health teams in promoting the quality of dying and death. Future studies should be conducted to evaluate funeral ritual preferences in countries with cultures similar to Brazil.
